# Cecal Ischemia Requiring Ileocecectomy Following Endovascular Aneurysm Repair: A Case Report

**DOI:** 10.7759/cureus.110140

**Published:** 2026-06-02

**Authors:** Michael K Zickler, Yunus B Tekin, Joseph Cuppari

**Affiliations:** 1 General Surgery, McLaren Macomb, Mount Clemens, USA; 2 Vascular Surgery, McLaren Macomb, Mount Clemens, USA

**Keywords:** aaa - abdominal aortic aneursym, emergency exploratory laparotomy, endovascular repair of, endovascular repair of abdominal aneurysm, ileocecectomy, ischemic colitis, portal venous gas

## Abstract

Cecal ischemia following endovascular aneurysm repair (EVAR) is an exceptionally rare complication. To our knowledge, isolated cecal ischemia requiring major resection has not been previously documented as a distinct case report in the PubMed-indexed English-language literature. Here, we present the case of a 67-year-old male who underwent EVAR for an abdominal aortic aneurysm (AAA). After stent-graft deployment, he had a patent superior mesenteric artery. He was discharged home on postoperative day 1. On postoperative day 2, he presented to an outside hospital with groin and abdominal pain, and imaging revealed cecal pneumatosis. He underwent emergent exploratory laparotomy and ileocecectomy with primary anastomosis. Pathology demonstrated transmural ischemic colitis.

## Introduction

Bowel ischemia is a rare but serious complication following abdominal aortic aneurysm (AAA) repair. Its incidence is significantly lower after endovascular aneurysm repair (EVAR) compared with open repair, with reported rates of 0.6% to 0.9% following EVAR versus 3.6% to 7.6% after open repair [1-3]. Despite its infrequency, postoperative bowel ischemia is associated with substantial morbidity and mortality, making early recognition critical.

Most reported cases of bowel ischemia following EVAR involve the left colon [4]. In contrast, isolated cecal ischemia after EVAR is exceedingly uncommon and, to our knowledge, has not been previously described in the literature. This case report describes a 67-year-old man who developed acute cecal ischemic colitis requiring emergent ileocecectomy with primary anastomosis shortly after EVAR for an AAA, highlighting a rare and atypical postoperative complication.

## Case presentation

A 67-year-old man with a past medical history significant for hypertension, hyperlipidemia, chronic obstructive pulmonary disease, coronary artery disease status post coronary artery bypass grafting, former tobacco use, and bilateral lower extremity claudication with activity status post left femoral endarterectomy underwent outpatient evaluation for an AAA. Preoperative computed tomography angiography (CTA) of the abdomen and pelvis demonstrated a 6.1-cm infrarenal AAA involving the mid to distal abdominal aorta, as well as circumferential calcifications of the superior mesenteric artery (SMA), estimated at approximately 50% stenosis (Figure 1). No preoperative abdominal symptoms were reported.

**Figure 1:**
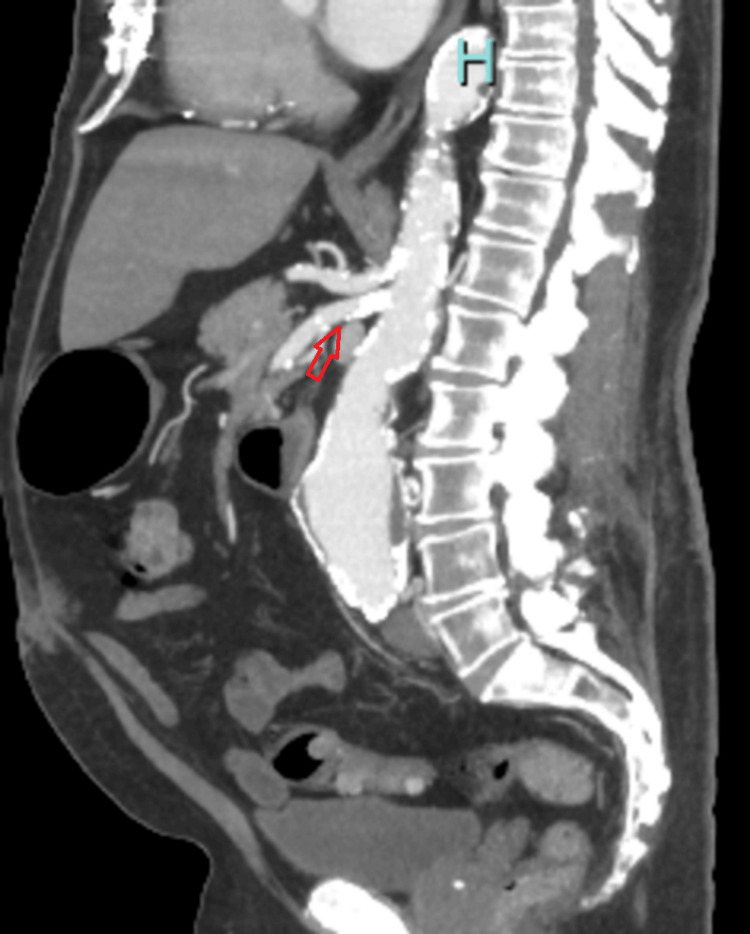
Preoperative CTA of the abdomen and pelvis, sagittal view, demonstrating a patent celiac trunk and superior mesenteric artery (SMA). Calcifications are present at the SMA origin (red arrow). CTA: Computed tomography angiography.

The patient subsequently underwent elective EVAR without intraoperative complications. There was no documented hypotension throughout the case. Completion angiography demonstrated appropriate graft positioning with preserved flow through the visceral vessels, including a patent SMA. The patient’s postoperative course was initially uncomplicated. He was tolerating a regular diet, denied abdominal pain, and was discharged home on postoperative day (POD) 1.

On POD 2, the patient presented to an outside hospital with acute onset of severe right groin pain, generalized abdominal pain, and nausea that began the evening prior. He reported having a normal bowel movement without melena or hematochezia. CTA of the abdomen and pelvis revealed pneumatosis and inflammation involving the cecum (Figure 2), concerning for bowel ischemia. The patient was also found to have portal venous gas (Figure 3). The previously noted SMA calcifications and patency were visualized (Figure 4). There was no evidence of endoleak or graft migration.

**Figure 2:**
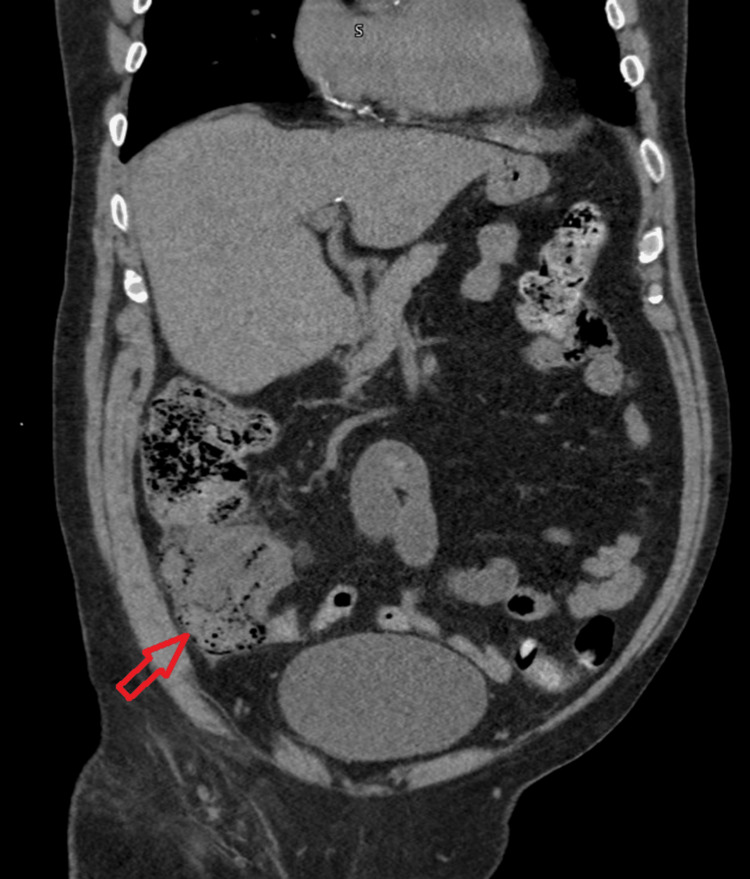
CTA of the abdomen and pelvis, coronal view, on postoperative day 2 demonstrating cecal pneumatosis and inflammation (red arrow). CTA: Computed tomography angiography.

**Figure 3:**
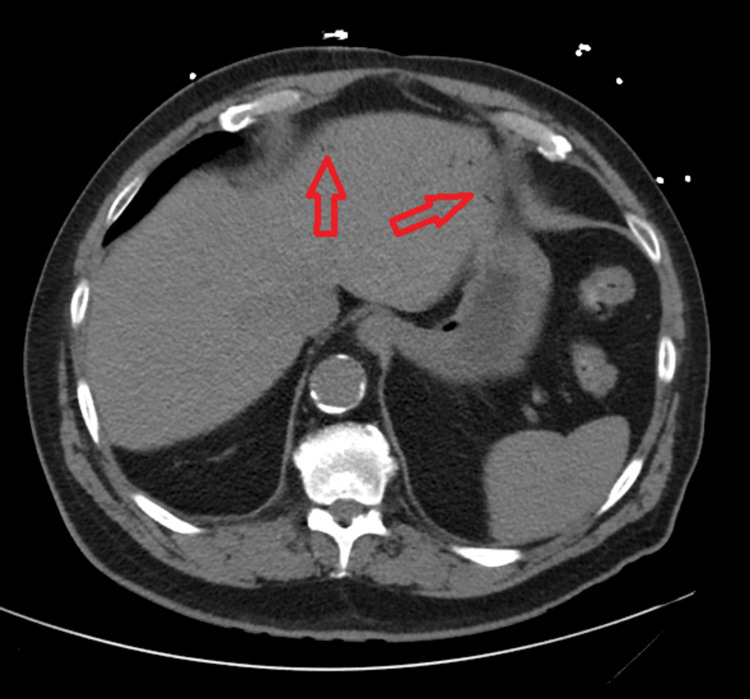
CTA of the abdomen and pelvis, axial view, on postoperative day 2 demonstrating portal venous gas (red arrows). CTA: Computed tomography angiography.

**Figure 4:**
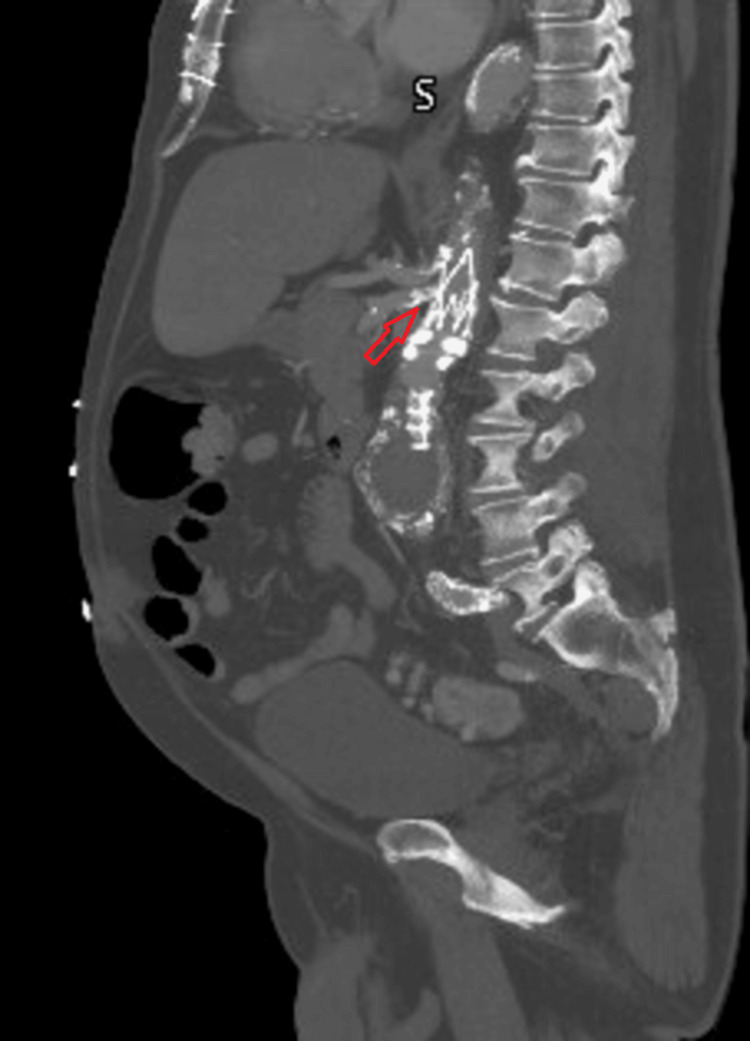
CTA of the abdomen and pelvis, sagittal view, on postoperative day 2 demonstrating a patent superior mesenteric artery (SMA) with an aortic stent graft in place (red arrow). CTA: Computed tomography angiography.

Given the imaging and physical examination findings concerning for acute bowel ischemia, the patient was taken emergently to the operating room for exploratory laparotomy. Intraoperative findings were notable for ischemia of the cecum, and an ileocecectomy with primary anastomosis was performed. A side-to-side functional end-to-end antiperistaltic anastomosis was performed using multiple staple loads. Pathologic examination of the resected specimen demonstrated hemorrhagic ischemic colitis with severe transmural ischemic colitis and acute serositis. All examined sections were negative for dysplasia or invasive malignancy. The terminal ileum and appendix were benign. There was no mention of cholesterol crystals or microthrombi in the report. While still an inpatient at the outside hospital, the patient developed a small bowel obstruction due to fascial dehiscence. He underwent exploratory laparotomy, abdominal washout, and primary closure of the fascia. The ileocolonic anastomosis was examined and found to be viable. He was eventually discharged 14 days after his original exploratory laparotomy with ileocecectomy.

## Discussion

Bowel ischemia remains a rare but devastating complication following AAA repair and, although less common after EVAR, is associated with substantial morbidity and mortality when it occurs [1-4]. Although EVAR avoids prolonged aortic cross-clamping and global hypoperfusion, ischemic colitis remains a recognized postoperative risk due to mechanisms distinct from those seen in open repair. When bowel ischemia develops after EVAR, it most commonly involves the left colon and is typically associated with inferior mesenteric artery compromise, hypogastric artery occlusion, or embolic phenomena [5-8].

The pathophysiology of bowel ischemia following EVAR is multifactorial [2]. Proposed mechanisms include transient hypoperfusion, disruption of collateral circulation, and embolization of atheromatous debris during device deployment [6-8]. Among these, microembolization has emerged as an important etiologic factor, with pathological studies demonstrating cholesterol crystal emboli and atheromatous material within resected bowel specimens [6,7]. In contrast to the more commonly described left-sided colonic ischemia, isolated cecal ischemia suggests compromise of the superior mesenteric or ileocolic arterial circulation rather than the inferior mesenteric artery. In the present case, preexisting SMA calcification and the close temporal relationship to EVAR raise concern for distal embolic compromise despite angiographic patency of the SMA at the time of repair.

Isolated cecal ischemia following EVAR is exceedingly uncommon. Most reported cases of post-EVAR bowel ischemia describe left-sided involvement, reflecting the vulnerability of watershed regions supplied by the inferior mesenteric artery [8]. In contrast, the cecum is typically protected by robust collateral circulation from the ileocolic artery, making ischemia in this region rare. However, embolization from thrombus or atheroma in the proximal aneurysmal neck may cause bowel ischemia, as evidenced by pathological findings of microembolization in resected specimens [7]. This case, therefore, expands the spectrum of bowel ischemia patterns that may occur following EVAR and highlights a potentially previously undescribed anatomic distribution.

Microembolization is a recognized mechanism of bowel ischemia following endovascular aneurysm repair, often resulting in segmental or patchy patterns of necrosis on pathological examination [7]. Accordingly, intraoperative imaging confirming patency of the major mesenteric vessels does not exclude downstream microembolic injury or impaired distal bowel perfusion [7]. As demonstrated in this case, bowel ischemia may present after an initially uncomplicated postoperative course, emphasizing the need for continued clinical vigilance in the early post-EVAR period [8,9]. Preoperative CTA provides a detailed assessment of aneurysm morphology and visceral vessel patency; however, postoperative ischemic complications are influenced by multiple patient- and procedure-related factors beyond imaging findings [10,11].

Management of bowel ischemia following EVAR depends on the severity and extent of involvement, with prompt recognition and early intervention being critical, particularly in cases of transmural ischemia or perforation [1,3,12]. Patients who require operative management experience significantly higher morbidity and mortality, with reported 30-day mortality rates ranging from 34.6% to 40.5% [1-3]. Approximately half of patients with ischemic colitis ultimately require surgical intervention [3]. In this case, early surgical exploration and ileocecectomy were required, with pathological examination confirming severe transmural ischemic colitis.

## Conclusions

This case highlights an exceedingly rare presentation of acute cecal ischemic colitis following EVAR for an AAA. Although bowel ischemia after EVAR most commonly involves the left colon, this report demonstrates that isolated right-sided colonic ischemia can occur; we suspect this was likely related to embolic compromise of the superior mesenteric or ileocolic arterial circulation. Awareness of this atypical presentation is important, as bowel ischemia may develop despite an initially uncomplicated postoperative clinical course and patent major mesenteric vessels on imaging. Early recognition and prompt surgical intervention remain critical to optimizing outcomes in this potentially devastating complication.
